# Supplementary material for: Coherent heteroepitaxial growth of I-III-VI2 Ag(In,Ga)S2 colloidal nanocrystals with near-unity quantum yield for use in luminescent solar concentrators
Source: Nat Commun. 2023 Jun 24;14:3779. doi: 10.1038/s41467-023-39509-y (PMC10290649; doi:10.1038/s41467-023-39509-y)
Supplement: Supplementary file 1 — Supplementary Information [file 41467_2023_39509_MOESM1_ESM.pdf]

Supplementary Information

Coherent Heteroepitaxial Growth of I-III-VI_2 Ag(In,Ga)S_2 Colloidal Nanocrystals with Near-unity Quantum Yield for Use in Luminescent Solar Concentrators

Hak June Lee[1,2†], Seongbin Im[1†], Dongju Jung[1†], Kyuri Kim[1], Jong Ah Chae[1], Jaemin Lim[1], Jeong Woo Park[1], Doyoon Shin[1], Kookheon Char[2], Byeong Guk Jeong[3], Ji-Sang Park[1], Euyheon Hwang[1], Doh C. Lee[4], Young-Shin Park[5], Hyung-Jun Song[6], Jun Hyuk Chang[7*], Wan Ki Bae[1*]*

[1]SKKU Advanced Institute of Nanotechnology (SAINT), Sungkyunkwan University (SKKU), Suwon 16419, Republic of Korea

[2]School of Chemical and Biological Engineering, Seoul National University, Seoul 08826, Republic of Korea

[3]School of Chemical and Biomolecular Engineering, Pusan National University, Busan 46241, Republic of Korea

[4]Department of Chemical and Biomolecular Engineering, KAIST Institute for the Nanocentury, Korea Advanced Institute of Science and Technology (KAIST), Daejeon 34141, Republic of Korea

[5]Chemistry Division, Los Alamos National Laboratory, Los Alamos, NM 87545, USA

[6]Department of Safety Engineering, Seoul National University of Science and Technology, Seoul 01811, Republic of Korea

[7]Department of Chemistry, James Franck Institute, and Pritzker School of Molecular Engineering, University of Chicago, Chicago, Illinois 60637, United States

†These authors contributed equally to this work.

Correspondence to Wan Ki Bae (wkbae@skku.edu); Jun Hyuk Chang (junhyuk@uchicago.edu); Hyung-Jun Song (hj.song@seoultech.ac.kr)

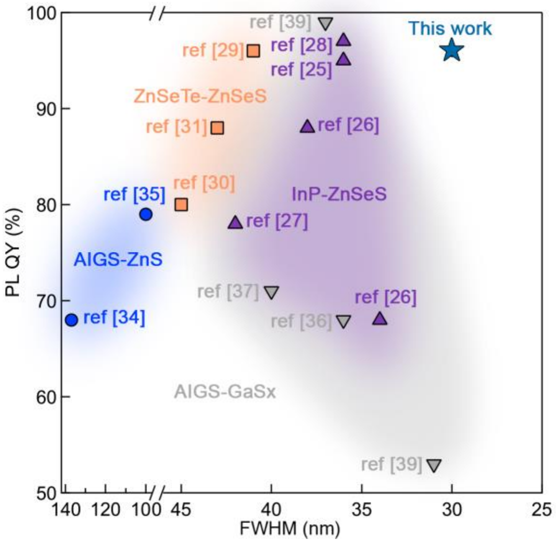

Supplementary Fig. 1 | Optical properties of heavy metal-free QDs in previous literatures. PL QY and FWHM comparison among heavy metal-free QDs. Detailed data are listed in Table 1.

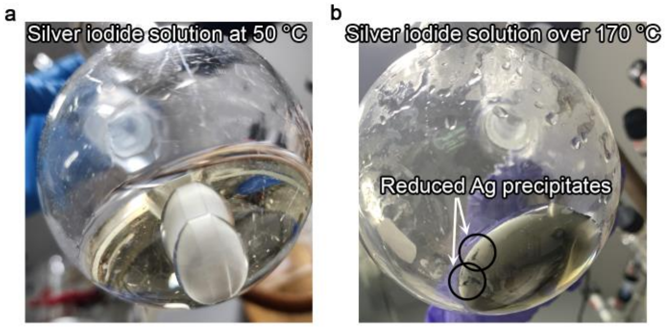

Supplementary Fig. 2 | Stability of Ag^{+} ion. Photographic images of silver iodide solution (a) at 50 °C and (b) over 170 °C. Black precipitates is reduced Ag.

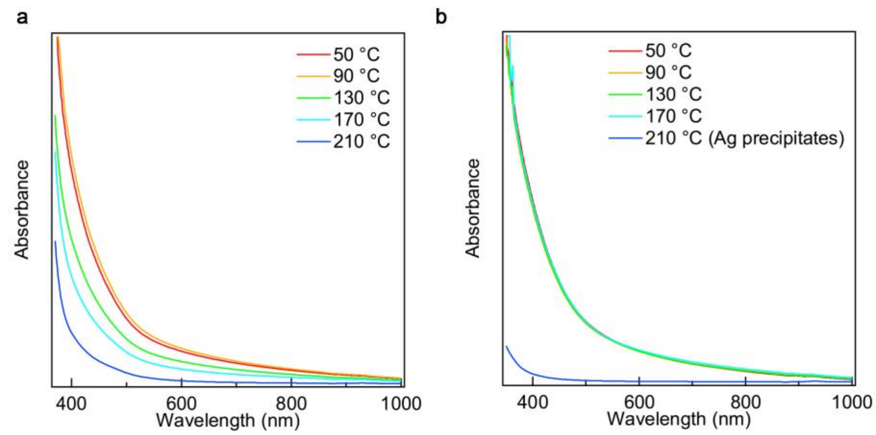

Supplementary Fig. 3 | Role of H_2S at Ag_2S NPs dissolution. Absorption spectra of Ag_2S NPs mixed with S-OAm vacuumed at (a) RT versus (b) 130 °C (H_2S removed) under varying temperatures. Ag_2S NPs are gradually dissolved into AgSH with S-OAm degassed at RT, whereas Ag_2S NPs remain unchanged in case with S-OAm degassed at 130 °C.

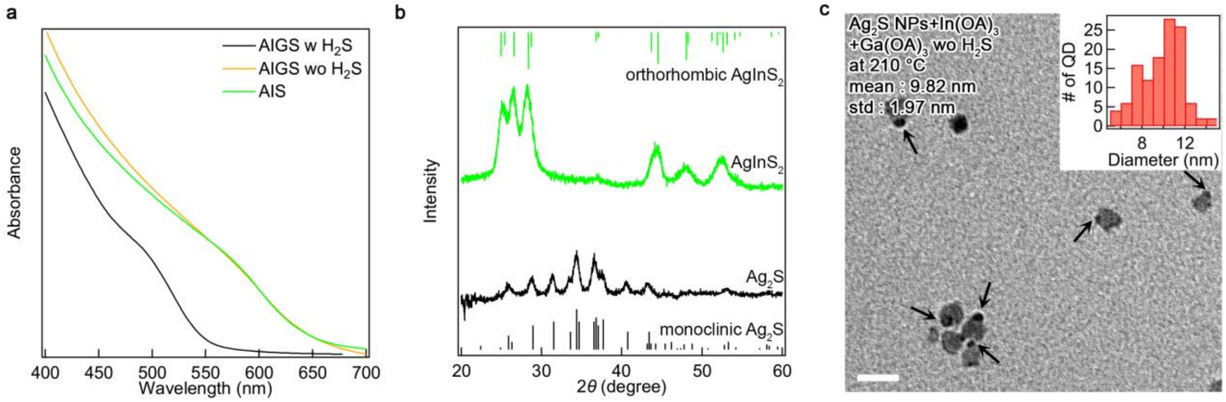

Supplementary Fig. 4 | Role of H_2S at AIGS cores nucleation. (a) Absorption spectra of NCs obtained from the mixed solution of Ag_2S NPs, $In(OA)_3$ and $Ga(OA)_3$ with (black) versus without in-situ generated H_2S (orange). Absorption spectrum of AIS NCs (green) obtained from the mixed solution of Ag_2S NPs and $In(OA)_3$ is shown for comparison. The Ga precursor is activated only with the presence of in-situ generated H_2S. (b) XRD patterns of AIS and Ag_2S NPs. (c) TEM image of Ag_2S NPs+$In(OA)_3$+$Ga(OA)_3$ without H_2S. Small AIS islands (marked with arrows) appear on Ag_2S NPs, which shows the structural transformation from Ag_2S NPs to AIS NCs through the cation exchange process.

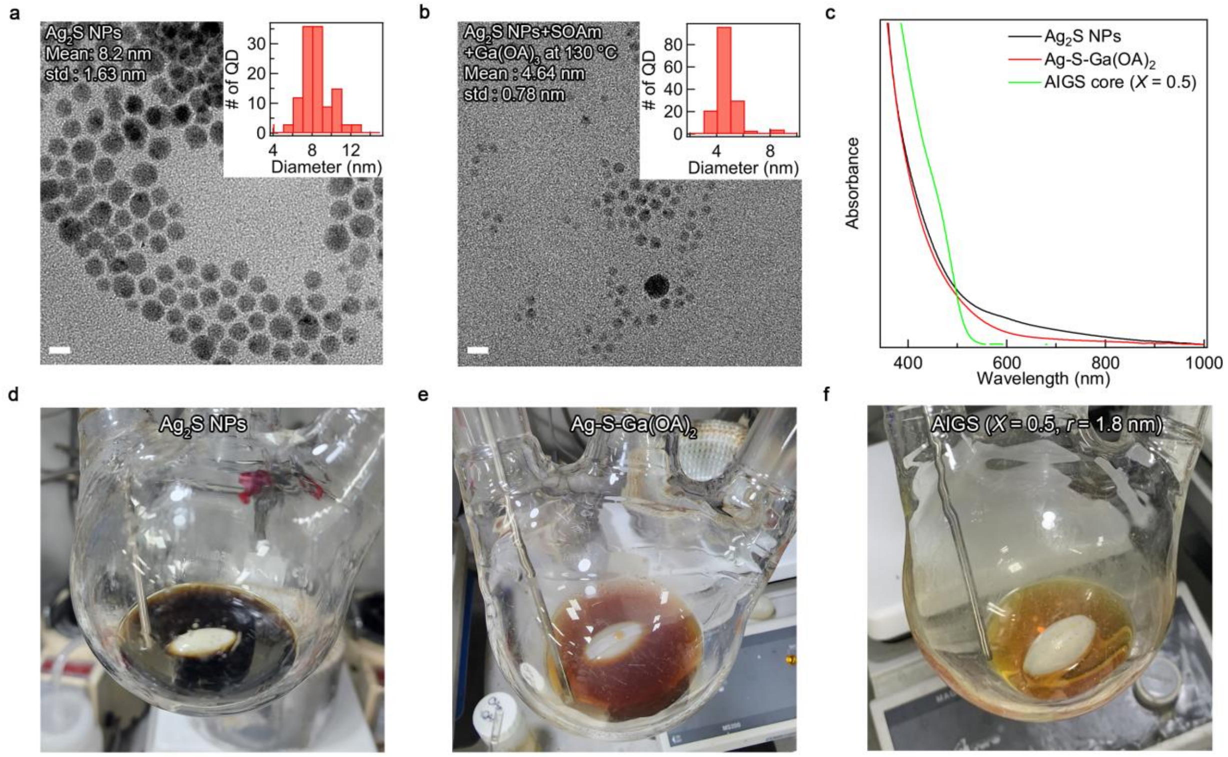

Supplementary Fig. 5 | Formation of Ag-S-Ga(OA)$_2$. TEM images of (a) Ag_2S NPs and (b) Ag_2S NPs with additional S-OAm and $Ga(OA)_3$ at 130 °C (scale bars = 50 nm). The insets in (a) and (b) show size distribution of Ag_2S NPs. (c) Absorption spectra of Ag_2S NPs (black), Ag-S-Ga(OA)$_2$ complex (red) and AIGS (X = 0.5, r = 1.8 nm) cores (green). Photographic images of (d) Ag_2S NPs, (e) Ag-S-Ga(OA)$_2$ complex, and (f) AIGS (X = 0.5, r = 1.8 nm) cores.

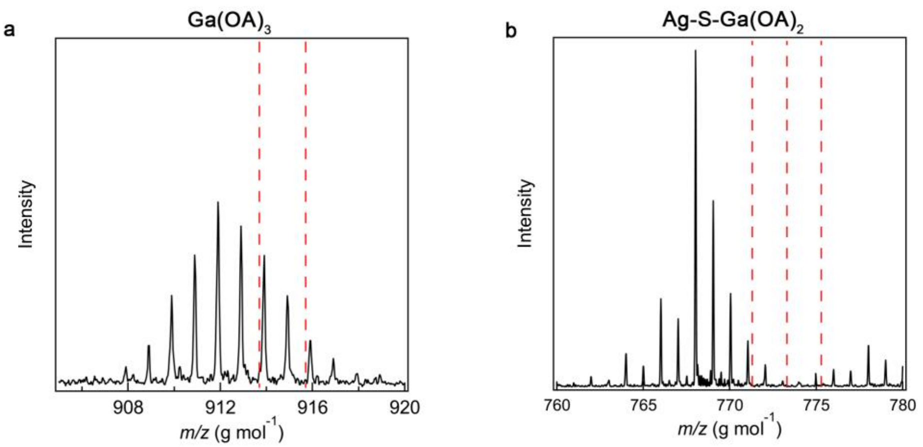

Supplementary Fig. 6 | Analysis on deviation in Ag-S-Ga(OA)$_2$ mass spectrometry result. MALDI-TOF mass spectra of (a) Ga(OA)$_3$ and (b) Ag-S-Ga(OA)$_2$. Dashed red lines in (a) and (b) represent theoretical *m/z* value from Ag and Ga isotopes. The slight deviation from theoretical *m/z* value of Ag-S-Ga(OA)$_2$ is likely originated from Ga(OA)$_3$, whose measured values also deviate from theoretical value.

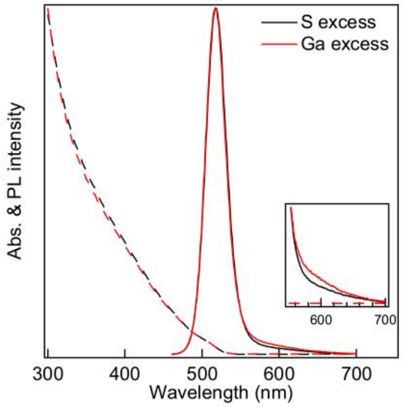

Supplementary Fig. 7 | Comparative advantages of S rich reaction condition. Absorption and PL spectra of AIGS (X = 0.5, r = 1.8 nm) -AGS (l = 1.6 nm) NCs synthesized from S rich reaction condition (Ag:Ga:S = 1:4.5:10, black line) versus Ga rich reaction condition (Ag:Ga:S = 1:4.5:4.5, red line). The inset shows the magnified view of tail emission in PL spectra.

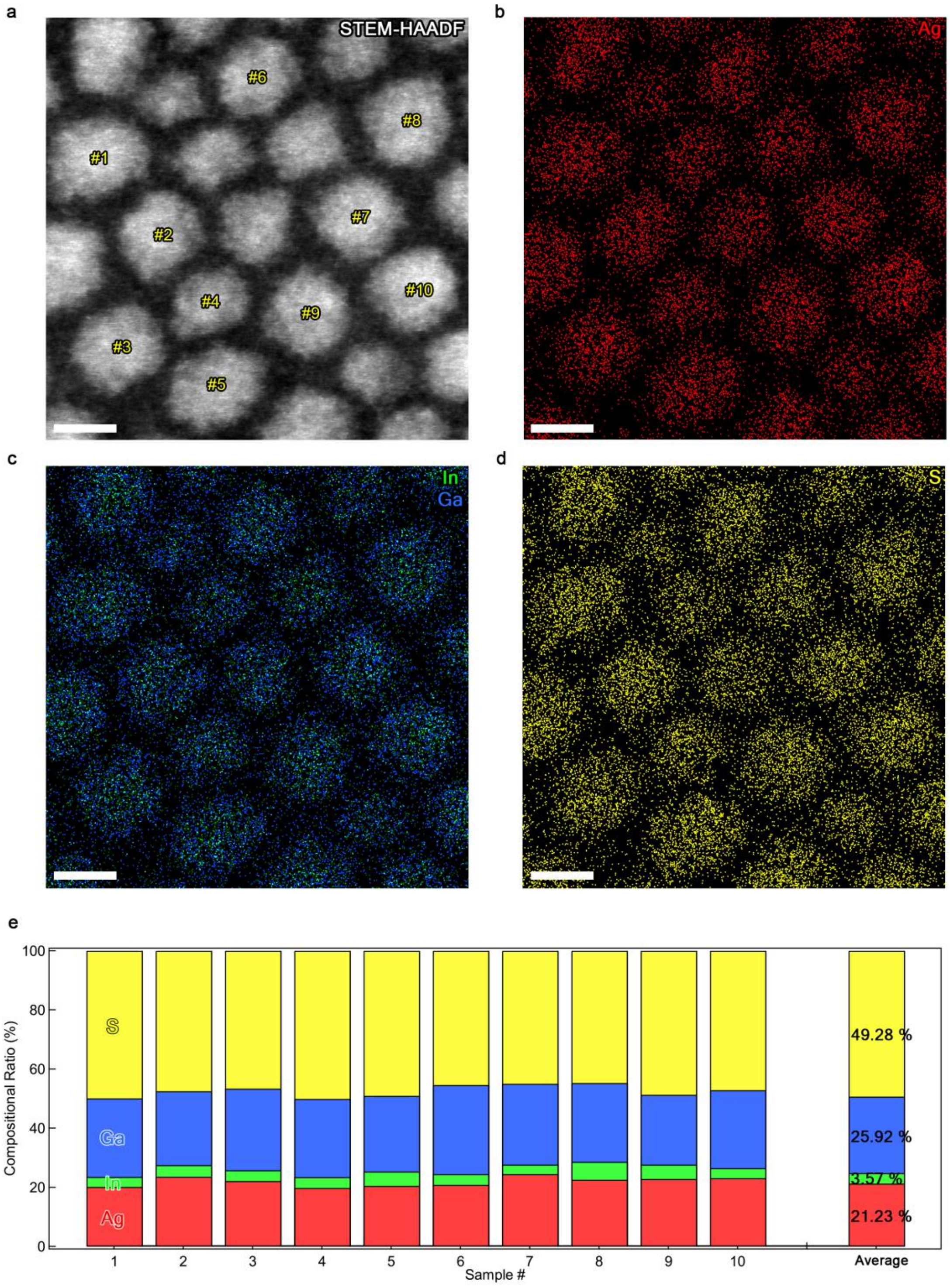

Supplementary Fig. 8 | Compositional homogeneity in individual NCs. (a-d) STEM-HAADF image and EDS elemental mapping of Ag (red), In (green), Ga (blue) and S (yellow) for AIGS (X = 0.5, r = 1.8 nm)-AGS (l = 1.2 nm) NCs. (e) Composition ratios of individual AIGS (X = 0.5, r = 1.8 nm)-AGS (l = 1.2 nm) NC described in the Supplementary Fig. 8a. Scale bars are 5 nm.

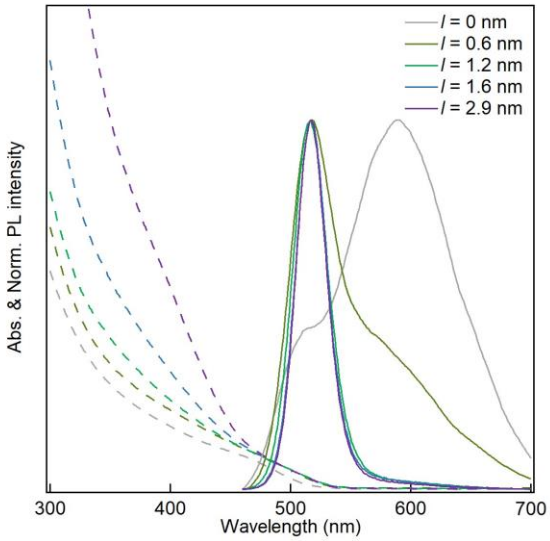

Supplementary Fig. 9 | Optical properties of AIGS-AGS NCs with varying shell dimensions. 2D plot UV-Vis and PL spectra of AIGS (X = 0.5, r = 1.8 nm)-AGS NCs with varying shell thicknesses ($0 \leq l \leq 2.$ 9 nm).

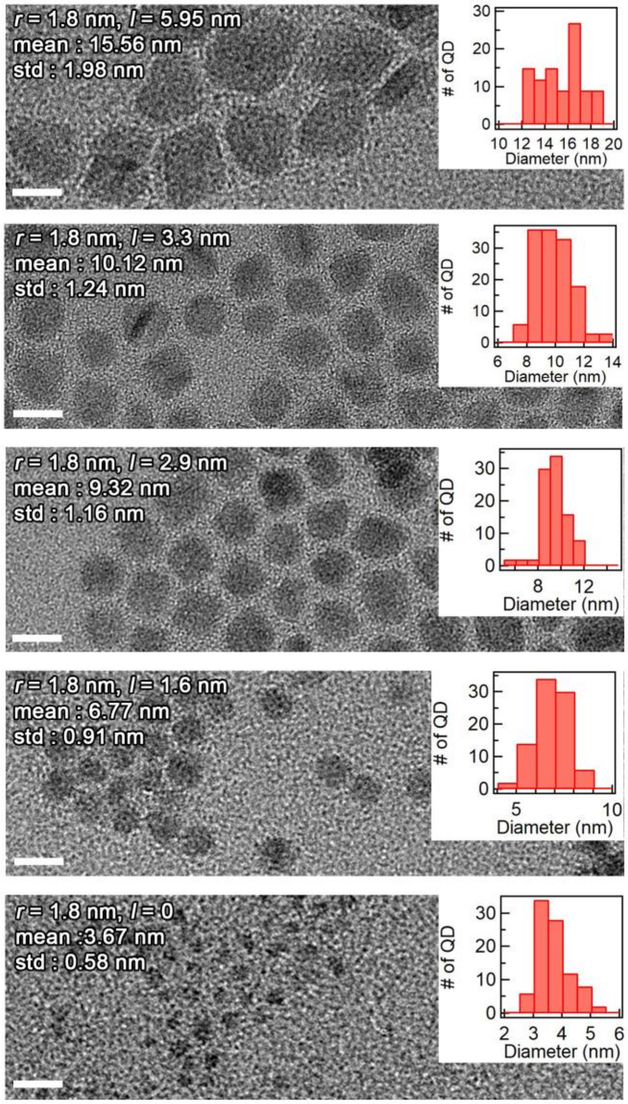

Supplementary Fig. 10 | Size analysis on AIGS-AGS NCs with varying shell dimensions. TEM images of AIGS (X = 0.5, r = 1.8 nm)-AGS NCs with varying AGS shell thicknesses ($0 \leq l \leq 5.95$ nm). Scale bars are 10 nm. Insets show the size distributions.

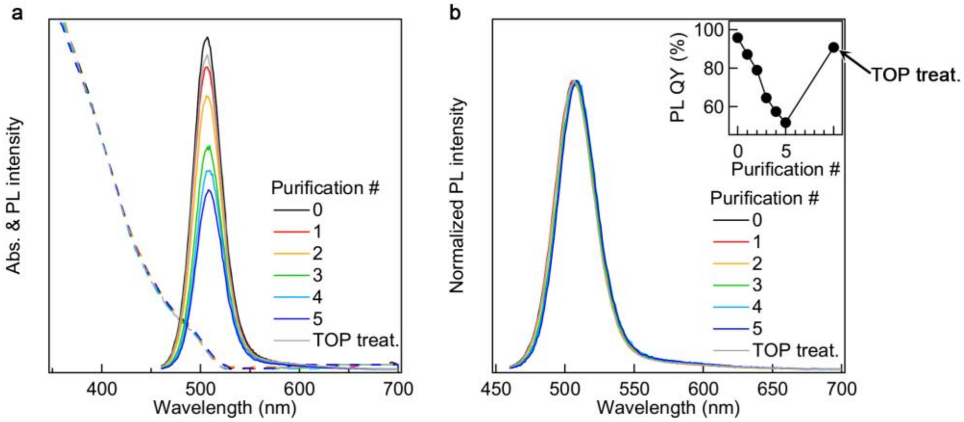

Supplementary Fig. 11 | Optical properties of AIGS-AGS NCs depending on purification steps. (a) Absorption, PL spectra and (b) normalized PL spectra of AIGS (X = 0.5, r = 1.8 nm)-AGS (l = 1.6 nm) NCs as a function of purification steps (precipitation with ethanol and redispersion with toluene). The inset of (b) shows PL QY of AIGS (X = 0.5, r = 1.8 nm)-AGS (l = 1.6 nm) NCs as a function of purification steps. Addition of TOP into 5-times purified AIGS-AGS NC solution aids to recover PL QY to its initial value.

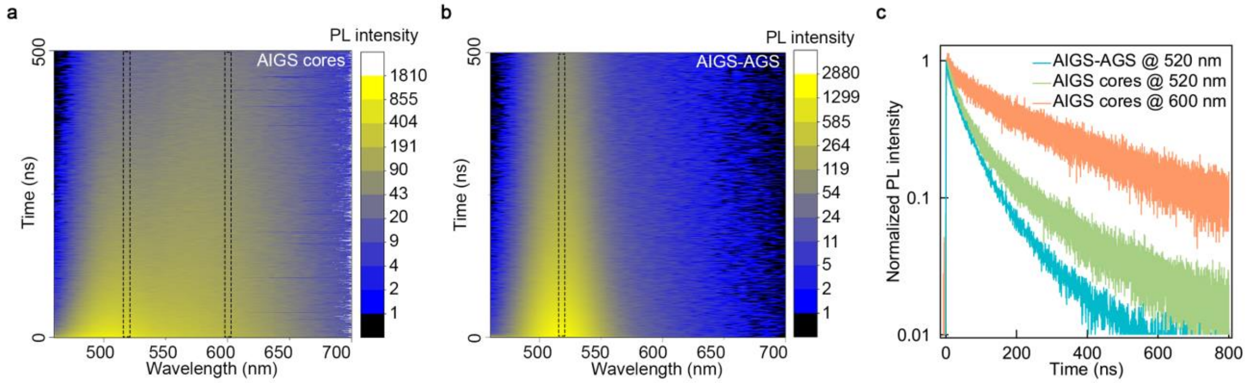

Supplementary Fig. 12 | Photophysical Properties of AIGS-AGS NCs. Time-resolved PL spectra of (a) AIGS (X = 0.5, r = 1.8 nm) cores and (b) AIGS (X = 0.5, r = 1.8 nm)-AGS (l = 1.6 nm) NCs. (c) PL decay curves at 520 nm and 600 nm.

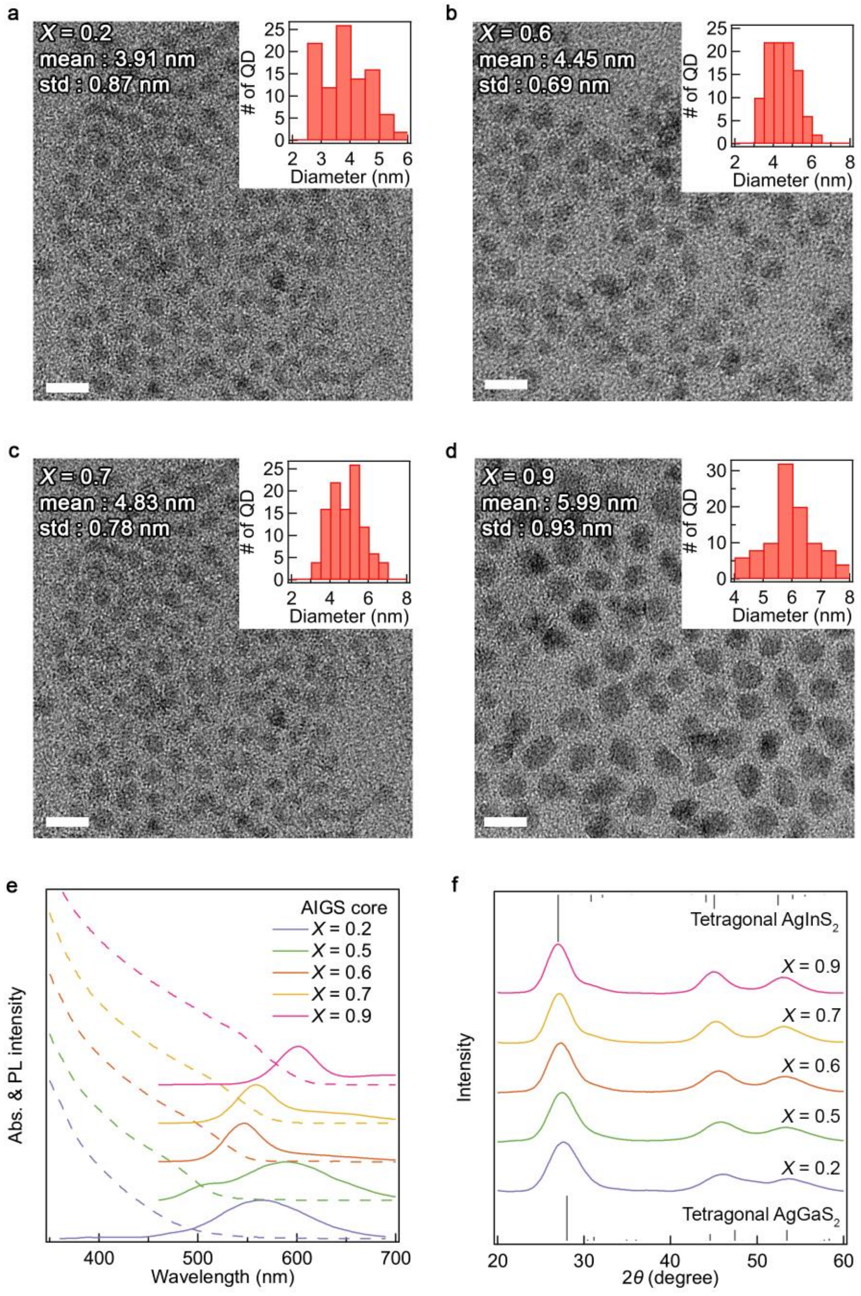

Supplementary Fig. 13 | AIGS cores with varying In contents. (a-d) TEM images (scale bars = 10 nm), (e) absorption and PL spectra and (f) XRD patterns of AIGS cores with varying In ratios ($0.2 \leq X \leq 0.9$). Spectra in (e) and XRD patterns of (f) are vertically shifted for visual clarity.

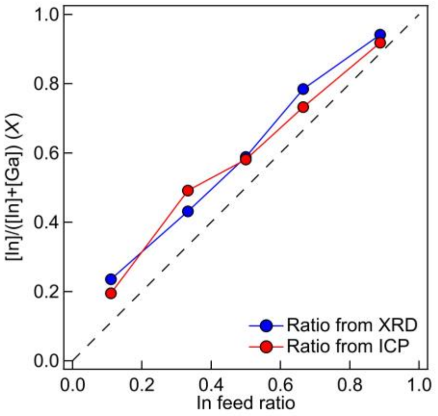

Supplementary Fig. 14 | Analysis on In contents (*X*) in AIGS cores. In contents (*X*) in AIGS cores versus In feed ratios. In contents (*X*) are obtained from ICP-AES (red) and estimated from XRD (blue).

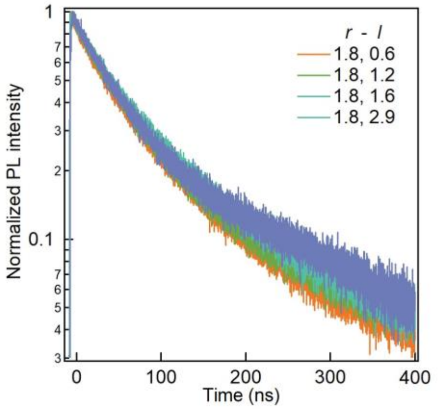

Supplementary Fig. 15 | Photophysical properties of AIGS-AGS NCs with varying AGS shell thicknesses. PL decay dynamics of AIGS (X = 0.5, r = 1.8 nm)-AGS NCs with varying AGS shell thicknesses (0.6 nm ≤ l ≤ 2.9 nm). The recombination characteristic times are similar regardless of shell thicknesses, indicating that AIGS-AGS NCs are in Type I potential profile.

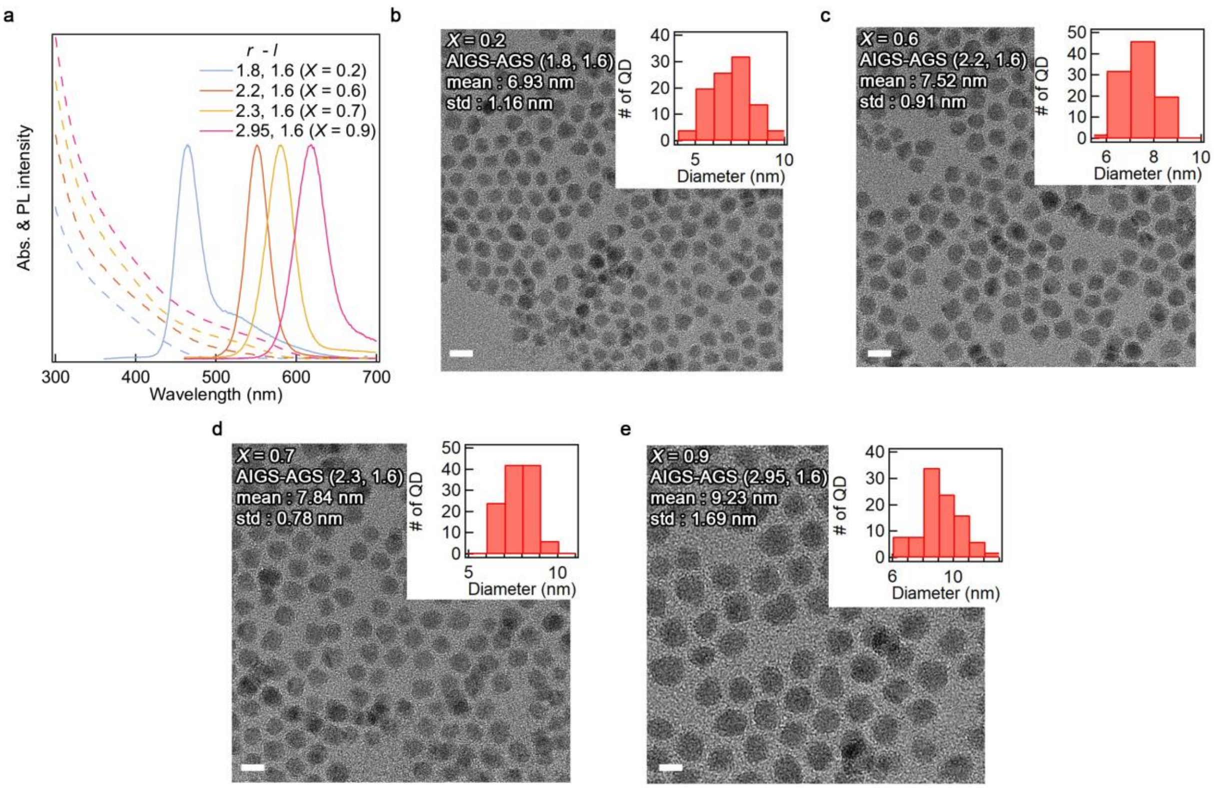

Supplementary Fig. 16 | AIGS-AGS NCs with varying core compositions. (a) Absorption and PL spectra, and (b-e) TEM images of AIGS-AGS NCs with varying core compositions ($0.2 \leq X \leq 0.9$) having the same shell thickness (l = 1.6 nm) (scale bars = 10 nm).

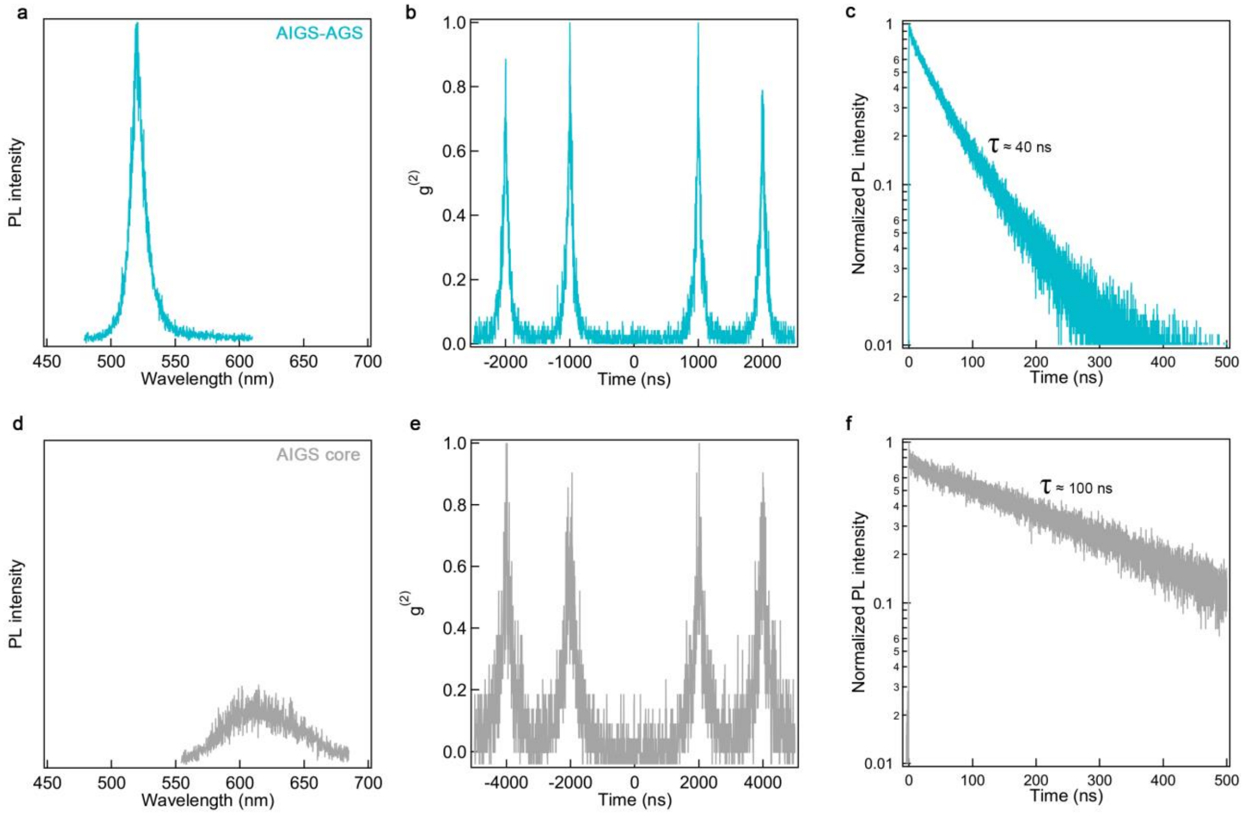

Supplementary Fig. 17 | Individual NC characterization. Individual NC photophysical characteristics of AIGS (X = 0.5, r = 1.8 nm)-AGS (l = 1.6 nm) NC and AIGS core (X = 0.5, r = 1.8 nm) (a,d) PL spectrum, (b,e) photon-photon correlation function with $g^{(2)}(0)$ = 0.014 and 0.005, respectively, and (c,f) PL decay curves.

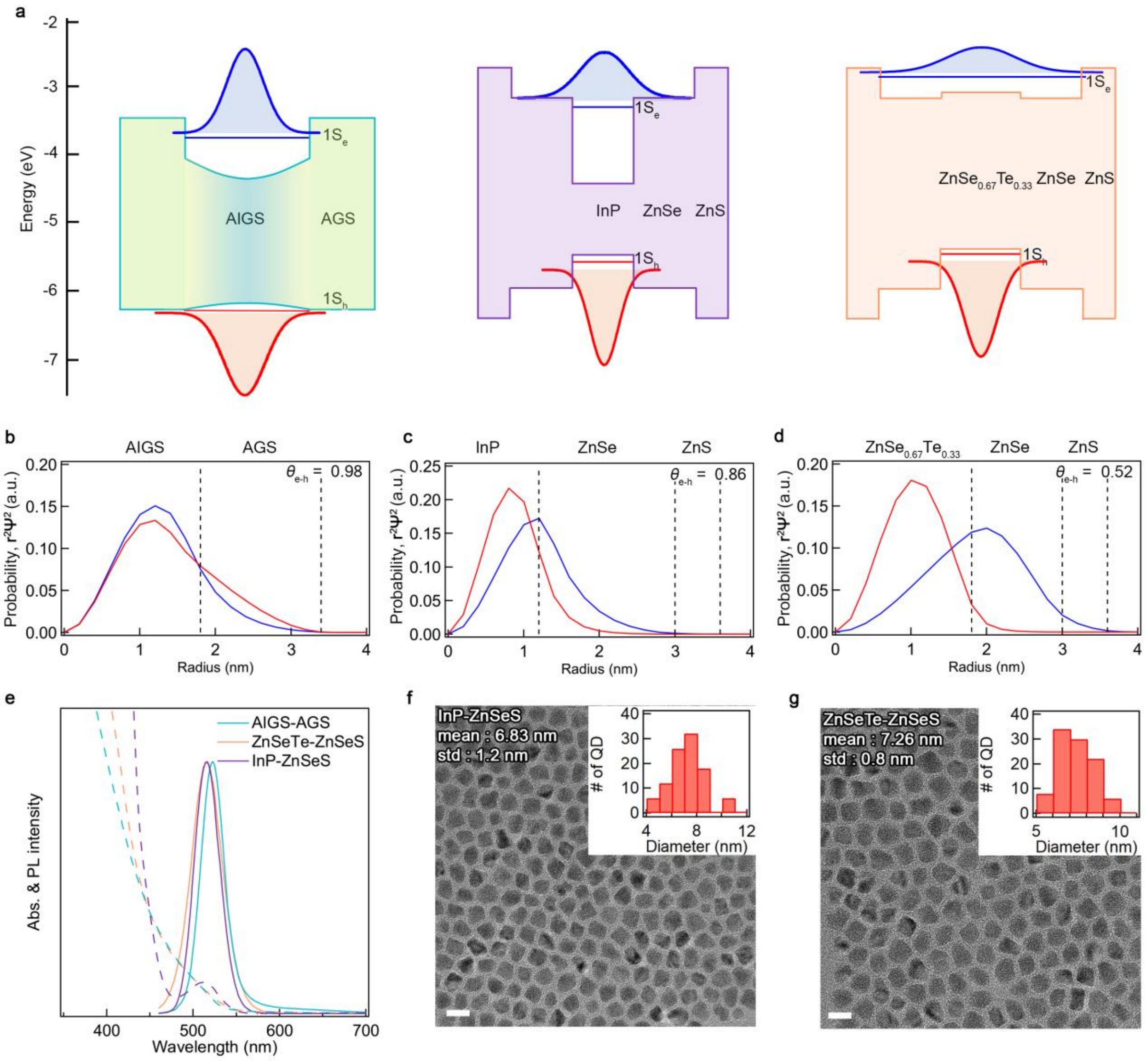

Supplementary Fig. 18 | Comparison of AIGS-AGS NCs with state-of-the-art heavy metal-free NCs. (a) Schematics illustrating potential profiles and electron (blue solide line) and hole (red solid line) wavefunctions at their lowest quantized energy state, (b-d) radial probability of electron and hole wavefunctions, and (e) absorption and PL spectra of AIGS (X = 0.5, r = 1.8 nm)-AGS (l = 1.6 nm), InP-ZnSeS (InP (r = 1.2 nm)-ZnSe (l = 1.8 nm)-ZnS (h = 0.5 nm) and ZnSeTe-ZnSeS ($ZnSe_{0.67}Te_{0.33}$ (r = 1.8 nm)-ZnSe (l = 1.2 nm)-ZnS (h = 0.6 nm)) NCs. TEM images of (f) InP-ZnSeS and (g) ZnSeTe-ZnSeS NCs. Scale bars = 10 nm.

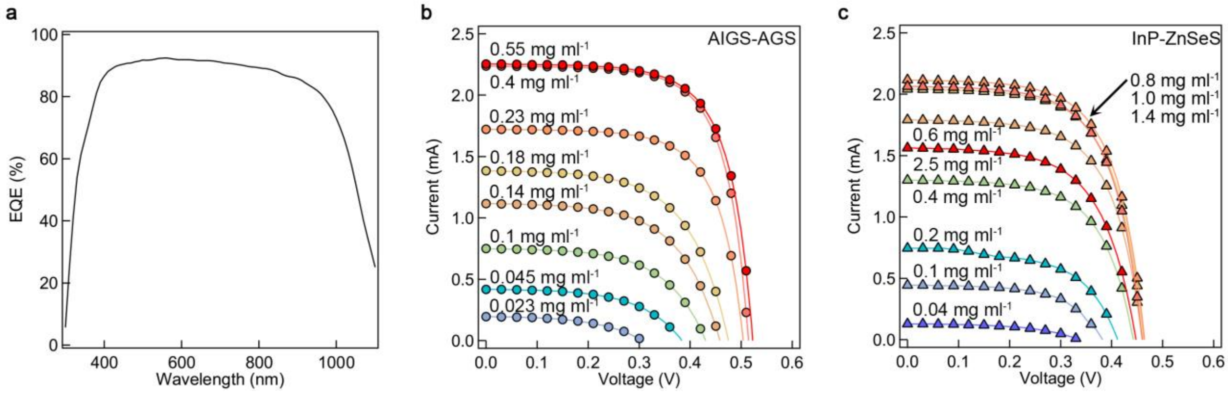

Supplementary Fig. 19 | Current-voltage characteristics of photovoltaic cell mounted at the edge of LSCs. (a) External quantum efficiency (EQE) spectrum of c-Si module. Current-voltage characteristics of edge-mounted c-Si cells with varying concentrations of (b) AIGS-AGS NC solutions and (c) InP-ZnSeS NC solutions under 420 nm LED irradiation.

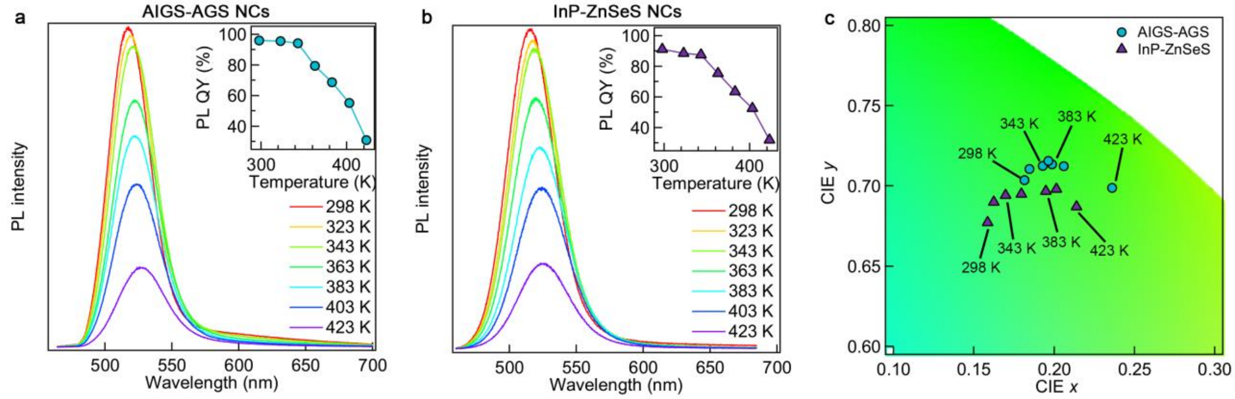

Supplementary Fig. 20 | Temperature effect on the emission properties. Temperature dependent (a,b) PL spectra of (a) AIGS (X = 0.5, r = 1.8 nm)-AGS (l = 1.6 nm) and (b) InP-ZnSeS (InP (r = 1.2 nm)-ZnSe (l = 1.8 nm)-ZnS (h = 0.5 nm)) NCs and (c) their color coordinates. The insets of (a) and (b) show temperature dependent PL QYs. The marked lines indicate corresponding temperature.

Supplementary Table 1 | Bond dissociation energy. Calculated bond dissociation energies from $A(OA)_3$ to $[A(OA)_2]^+$ ($A = In^{3+}$, Ga^{3+}, Al^{3+}).

	Bond dissociation energy (eV)
$Al(OA)_3$	4.87
$Ga(OA)_3$	3.25
$In(OA)_3$	2.59
